# Dimensions of Hallucinations and Delusions in Affective and Nonaffective Illnesses

**DOI:** 10.1155/2013/616304

**Published:** 2013-08-13

**Authors:** Ranju Kumari, Suprakash Chaudhury, Subodh Kumar

**Affiliations:** ^1^Ranchi Institute of Neuro-Psychiatry and Allied Sciences, Ranchi, Jharkhand 834006, India; ^2^Department of Psychiatry, Pravara Institute of Medical Sciences (Deemed University), Rural Medical College & Hospital, District Ahmednagar, Loni, Maharashtra 413736, India

## Abstract

The aim of the study was to examine the dimensions of hallucinations and delusions in affective (manic episode, bipolar affective disorder, and depressive episode) and nonaffective disorders (schizophrenia, acute and transient psychotic disorders, and unspecified psychosis). Sixty outpatients divided equally into two groups comprising affective and nonaffective disorders were taken up for evaluation after screening, as per inclusion and exclusion criteria. Scores of 3 or above on delusion and hallucinatory behavior subscales of positive and negative syndrome scale were sufficient to warrant rating on the psychotic symptom rating scales with which auditory hallucination and delusion were assessed on various dimensions. Insight was assessed using the Beck cognitive insight scale (BCIS). There were no significant differences between the two groups on age, sex, marital status, education, and economic status. There were significant differences in total score and emotional characteristic subscale, cognitive interpretation subscale, and physical characteristic subscale of auditory hallucination scales in between the two groups. Correlation between BCIS-total and total auditory hallucinations score was negative (Spearman Rho −0.319; *P* < 0.05). Hallucinating patients, more in nonaffective group, described a negative impact of hallucinating voices along with emotional consequences on their lives which lead to distress and disruption.

## 1. Introduction

Hallucinations may be viewed as incomprehensible experiences that the person describes or interprets, and that perception is accompanied by feelings, such as urgency, certainty, and vividness. Delusion is a false belief based on incorrect inference about external reality and its explanations are in conflict with the evidence. Both phenomena are often a cause of distress, preoccupation, and significant interference in daily functioning. Junginger and Frame [1] have argued that the important characteristic of voices perceived as outside the head is not their location per se but rather the person's delusional attribution that they are aliens. In this context, relations between hallucinations and delusions need to be examined more carefully. The majority of hallucinations are examples of secondary delusions since the person is always trying to interpret or make sense of the anomalous experiences and that leads to secondary delusions. Evidence of the coexistence of hallucinations and delusions suggests that these two symptoms may share common ground in terms of the psychological factors underlying their presence [2–5].

The experience of hallucination can entail a change to multiple realms of personal and environmental experience that are described in light of each person's personal, social, and cultural influences [6, 7]. Perhaps the most striking form of hallucination is auditory verbal hallucinations (AVHs) that classically take the form of one or more “voices” that talk to or about the patient. AVHs are a common symptom of schizophrenia and mood disorder and hence have been a particular focus of assessment research. Despite having been given such diagnostic weight in classificatory systems, AVHs can be difficult to fully assess in the clinical context. First, they are not fully captured by operational criteria as the experience of “hearing voices” varies greatly between individuals and may involve numerous disturbances of agency, autonomy, and the “stream of consciousness.” Second, they are associated with profound transformations of self-awareness that may be difficult to describe, which can lead to feelings of estrangement from common human experience and communication [8, 9]. Finally, most patients note how even the most common description of AVH as “voices” is a rather poor metaphor, which does not fully capture their experiential fact. The hypothesis that metacognitive beliefs may play a key role in auditory hallucinations was first put forward by Morrison et al. [3], and auditory hallucinations are the result of intrusive thoughts misattributed to an external source. Such misattribution is influenced by metacognitive beliefs, which may contribute to the conceptualisation of such thoughts as externally generated. The topography of the voices is Identity, Beliefs about Identity, Form, Content (Positive Content, Negative Content), Commands, Content and Beliefs, Location, and Impact of Voice Experiences. Several factors can contribute to the intrinsic plausibility of auditory hallucinations like the structured versus unstructured auditory hallucinations, external versus an internal origin, the locus [10], bilateral versus unilateral, time location related versus unrelated to the patient's thoughts [11], emotions or actions, phrases versus single words, multiple voices versus single voice [11], auditory hallucinations fitting versus not fitting with the patient's desires or fears, and last but not least interactive versus noninteractive voices. Emotional factors may play a significant role in the case of psychiatric hallucinations. Three aspects of emotional factors are emotional antecedents, emotional content, and emotional consequences. AVHs have often a negative, maladaptive quality [12], but some AVHs may not have a particular emotional content and some patients may even state that their voices serve an adaptive function or even pleasant [13–15]. Evidence of cultural variations has clinical implications. The clinician must also take into account a person's cultural background when assessing and treating hallucinations. Bentall [16] pointed out that failure to appreciate the cultural context may prevent clinicians from responding appropriately to the distress experienced by their patients. The assessment must include a detailed evaluation of the hallucinations as well as the contexts in which they appear and also the consequences for the person and their carers, family members, friends, and so forth.

Systematic study of the phenomenology of delusions is a relatively recent enterprise and many fundamental questions remain unanswered. Strauss [17] was the first to point out that delusions can be characterized along several dimensions that are largely independent of their content; he proposed the dimensions of conviction, preoccupation, external determinants, and implausibility (the last is actually content-related). His idea has been elaborated and operationalized by a number of investigators who have focused on a wide range of delusional characteristics [18–23]. As a group, these studies suggest that many of the proposed dimensions can be measured reliably and appear to constitute independent constructs. The main characteristics of delusion are implausibility, idiosyncrasy, conviction, and incorrigibility [24]. The process of diagnosing the delusion involves a comparison between the delusional statement and reality. Delusions can best be characterized multidimensionally, by factor-analytic studies reporting three or four dimensions, most usually conviction, preoccupation, distress, and disruption to behaviour [19, 22, 25]. Investigations that take a multidimensional approach suggest that different cognitive and emotional processes contribute to these different dimensions such that delusional conviction and distress result from different processes [26]. A few multidimensional assessment tools for delusions have been developed to explore the dimensions [19, 25, 27–30]. The studies had used the dimensional approach to explore the unitary or diverse nature of delusions by comparing their characteristics across diagnostic categories. Harrow et al. [22] found no significant differences on three dimensions (conviction, perspective, and emotional commitment) across diagnostic groups. Jorgensen and Jensen [23] showed similar patterns across diagnostic groups on five dimensions (conviction, extension, systematization, probability, and pressure) but did not subject their findings to tests of significance. During course of illness or therapy, dimensions of delusional experience can change independently [31]. Emotional processes make a distinct contribution to the development and persistence of positive symptoms according to multifactorial model of psychosis [5, 32, 33]. An association between emotional distress and severity of delusions has been reported [34].

A standardized approach to the assessment of symptom dimensions is essential to yield precise information reflecting dimensional change coinciding with treatment outcome. The psychotic symptom rating scale is one of the scales which take into account various phenomenological characteristics and better suited for repeated clinical monitoring [35]. Few efforts have been made, however, to use the dimensional approach to explore the unitary or diverse nature of delusions and hallucination by comparing their characteristics across diagnostic categories. So this study was conceived to explore and throw light on the difference in dimensions of auditory hallucinations and delusions in affective and nonaffective groups of patients.

## 2. Methodology

The present study is a hospital-based cross-sectional study. The patients from Outpatient Department of Ranchi Institute of Neuro-Psychiatry and Allied Sciences were recruited by purposive sampling method as per the inclusion and exclusion criteria. Written informed consent was obtained from each subject. The protocol was submitted to and approved by the institutional ethical committee.

### 2.1. Subjects

The sample size was 60 patients equally divided into affective and nonaffective groups. The patients in both groups were between 18 and 60 years of both sexes. The diagnosis of the patients in affective group was mania with psychotic symptoms (*n* = 7), bipolar affective disorder, current episode manic with psychotic symptoms (*n* = 20), bipolar affective disorder, current episode severe depression with psychotic symptoms (*n* = 1), bipolar affective disorder, current episode mixed (*n* = 1), and severe depressive episode with psychotic symptoms (*n* = 1). In the nonaffective group, the diagnosis was paranoid schizophrenia (*n* = 17), undifferentiated schizophrenia (*n* = 8), acute and transient psychotic disorders (acute schizophrenia like-psychotic disorder) (*n* = 1), other acute predominantly delusional psychotic disorders (*n* = 1), and unspecified nonorganic psychosis (*n* = 3). All the diagnoses were made according to the ICD-10 DCR [35] criteria. Patients with schizoaffective disorder were not included in the study. Patients were drug naïve or drug free. Drug free was defined as period of 4 weeks for all psychotropics and anti-Parkinsonism drugs. Drug free for depot antipsychotics was 8 weeks. The patients in both groups were free from any comorbid psychiatric condition including substance abuse or dependence (except nicotine and caffeine abuse and dependence), serious medical disorder, or neurological condition as assessed by history, examination, and laboratory investigations. Pregnant women and nursing mothers and those not consenting for study were excluded. A total of 5 patients were excluded according to the specified exclusion criteria. Three patients had the diagnosis of mental and behavioural disorders due to psychoactive substance use, psychotic disorder. The other two patients' diagnosis was organic mood disorder and organic hallucinosis. As in the inclusion criteria we have mentioned the diagnosis to be entertained and in those diagnoses only we got pure patients sample which had hallucination and delusion unaffected by other explainable factors. So the generalization of the results obtained is not going to be affected by these exclusions. The ratings on the various scales were done by one of us (Subodh Kumar) who was not aware of the subjects' diagnosis at the time he performed the ratings.

### 2.2. Tools

*Sociodemographic and Clinical Data Sheet.* A specially designed sociodemographic and clinical data sheet including age, sex, marital status, education, economic status, occupation, support system, premorbid adjustment, number of hospitalizations, type of schizophrenia, onset, total duration, course specifier, duration of treated and untreated psychosis, childhood illness, and family history was prepared for the study.

*Brief Psychiatric Rating Scale Expanded (BPRS) [36].* The BPRS consisting of 24 items is rated on seven-point severity scales. The rating is based upon the observation made by the rater during interview and subject verbal report. The BPRS is appropriate for evaluating baseline psychopathology, clinical outcome, and treatment response. The BPRS has not only been used in schizophrenia, but also been used successfully in geriatric studies, in depression, anxiety disorders, and even in eating disorders and autism. Total score ranges from 16 to 112. Higher score denotes severity of symptoms. 

*Positive and Negative Syndrome Scale (PANSS) for Schizophrenia (Delusion and Hallucinatory Behavior Items) [37].* The PANSS, widely used in research settings, has been developed specifically to assess psychopathology in patients with schizophrenia. This scale is based on the concept that schizophrenia has two distinct syndromes: a positive syndrome including features like delusions and hallucinations and a negative syndrome comprising features like social withdrawal and affective blunting. It has 30 items which are rated on a 7-point continuum (1 = absent, 7 = extreme). P1 and P3 items represent delusions and hallucinatory behavior, respectively. Ratings are generally based upon information relating to the past week, and scores are provided in separate clinical domains.

*The Psychotic Symptom Rating Scale [38, 39] (PSYRATS).* The PSYRATS is a 17-item multidimensional measure of delusions and hallucinations on which each item is measured on a 5-point scale ranging from 0 (not endorsing item) to 4 (fully endorsing item). Hallucination subscale consists of 11 items and delusions subscale consists of 6 items. Symptoms over the past week are rated, with higher scores representing greater severity. Excellent interrater reliability [39] and a good sensitivity to change [38] have been shown. Research into the psychometric properties of the scale indicates that it is valid to consider three subscales on the AHS. The subscales are emotional characteristics (amount of negative content, degree of negative content, amount of distress, and intensity of distress), cognitive interpretation (beliefs regarding origin of voices, disruption, control), and physical characteristics (frequency, duration, location, loudness). Similarly, a two-factor solution for the delusional subscale was reported with one cognitive interpretation factor (amount of preoccupation, duration of preoccupation, conviction and disruption) and one emotional characteristics factor (amount of distress and intensity of distress).

*The Beck Cognitive Insight Scale (BCIS) [40].* BCIS was developed to evaluate patients' self reflectiveness and their overconfidence in their interpretations of their experiences. It consists of a 15-item self-report questionnaire, a 9-item self-reflectiveness subscale, and a 6-item self-certainty subscale. The first component consisted of 9 items measuring objectivity reflectiveness and openness to feedback and has given the label self-reflectiveness. Under the umbrella of decision-making and resistance to feedback, 6 items were united in a second component of the scale, labeled self-certainty. High scores on the subscale self-reflectiveness and low scores on subscale self-certainty are considered as normal. A composite index of the BCIS reflecting cognitive insight was calculated by subtracting the score for the self-certainty scale from that of the self-reflectiveness scale; a score of 10 points or more signifies good cognitive insight. Respondents are asked to rate how much they agree with each statement by using a 4-point scale that ranges from 0 (do not agree at all) to 3 (agree completely). No time frame for the ratings is provided. The coefficient *α* for the self-reflectiveness scale was 0.68 and for self-certainty was 0.60 for the original sample.

### 2.3. Study Procedure

Patients were taken up for evaluation and scoring after screening as per inclusion and exclusion criteria. At least one hallucination and/or one active delusion must be present in the patient in both groups. Scores of 3 or above on delusion and hallucinatory behaviour subscales of the PANSS were sufficient to warrant rating on the PSYRATS with which the dimension of auditory hallucinations and delusions was assessed. The overall psychopathology was assessed using BPRS and insight was assessed using the BCIS.

## 3. Statistical Analysis

The collected data was analyzed with the help of appropriate parametric (Independent sample *t*-test) and nonparametric (Chi-square, Mann-Whitney *U*, and Spearman rho) statistical methods, using the Statistical Package for Social Sciences—Version 16.0 (SPSS 16.0). Analyses were performed with a significance level of <0.05 and a confidence level of 95%.

## 4. Results

Male subjects were the predominant ones in our sample (44 (74%)) compared to females (16 (26%)). A majority of the subjects (54 (90%)) belonged to rural background, hailed from families with low socioeconomic status (*n* = 41; 68.3%), and were married (*n* = 51; 85%). There were no inter-group differences in clinical characteristics at baseline (age, sex, marital status, education, occupation, economic status, duration of treated psychosis) but significant differences in age of onset, duration of illness, and duration of untreated psychosis (Table 1). There was significant difference in onset, course, number of episodes, and form of voice between the two groups (Table 2). Thirteen patients in affective group had 2nd person auditory hallucination and 18 patients in nonaffective group had 3rd person auditory hallucination which is characteristic of diagnosis of schizophrenia. Significant differences were seen in most of the items of hallucination and one item of delusion in our study (Tables 3 and 4). Most predominant pervasive mood in nonaffective group in our study was indifferent (*n* = 13) and the next was irritable (*n* = 9). In the affective group only in 16 patients pervasive mood was euphoric while in the remaining 14 patients the pervasive mood was irritable (*n* = 11), anxious (*n* = 2), and depressed (*n* = 1). Significant differences were found in BCIS total and self-certainty scale (Table 5). There was a significant negative correlation between BCIS-total score and total auditory hallucination score (Spearman Rho −0.319, *P* < 0.05) but not between BCIS-total score and total delusion score (Rho −0.253, *P* = not significant). 

## 5. Discussion

### 5.1. Subject Characteristics

A total of 60 patients divided equally were included in two groups. There were no significant differences among two groups regarding baseline characteristics (sex, religion, marital status, education, community, occupation, habitat, mother tongue, and economic status), and they are similar to earlier studies [39, 41]. Mean duration of illness in our sample was less compared to other studies [42, 43]. Duration of untreated psychosis was more in our sample compared to duration of treated psychosis. This may be the reflection of low socioeconomic status and poor education of patients and their guardians. The number of patients having episode in affective group was more compared to nonaffective group. In the non-ffective group, the patients mainly had first episode with mostly continuous course and had longer duration of illness compared to affective group. In addition, duration of untreated psychosis was more in nonaffective group. This could lead to formation of intense psychosis and their auditory hallucination distressing both in terms of amount and intensity.

### 5.2. Emotional Impact of Auditory Hallucination (AH)

In many studies the emotional impact of AHs on their hearers is assessed in a very simple fashion, often by means of a single question [44]. Typically, little regard is paid to the complexity of emotional response or to the possibility of mixed reactions. In our study, this limitation is overcome by using PSYRATS scale which has extensive items on emotional impact of AHs. Previous studies of the emotional or evaluative impact of AHs suggest that most respondents describe their voices as unpleasant, but for sizable minorities the voices are either not unpleasant or pleasant [12–15]. In our study also, there were significant differences between affective and nonaffective groups when rated on the majority of PSYRATS scale items. Mean scores of previous items of hallucination in our study were less compared to another study [45]. 

AHs occur frequently in patients with schizophrenia and are usually highly distressing and disabling [46]. Chadwick et al. [47] stress the importance of the ABC model for cognitive therapy of hallucinations: the hallucinations are the activating events, which engender cognitions, which in turn yield emotional distress and anger. Voice content that was linked with a nonsignificant person in the patient's life and voices that could have not been the patient's own voice or thoughts was perceived as more unpleasant [46, 48, 49]. The impact of auditory hallucinations is usually one of the most clinically significant aspects of the experience and is affected by the person's attributions for the experience, their perception of control over the voices, their emotional state, culture, prior social experience, and ability to resist commands or requests. Previous studies have established that patients with AVH misidentified their own speech more often when the words read were of a negative or derogatory content as opposed to neutral or positive. [50, 51] Oulis et al. [52] concluded that “usually their “voices'” content is hostile to the patient.” The beliefs patients hold concerning the content of the voices, that is, who produces the voices, what they intend, what will happen to the subject if he does not obey the voices, and so forth, can produce distress and disruption in the life of nonaffective disorder patients. Romme et al. [12] found that 93% of their sample of hallucinating patients described a negative impact of hallucinating voices on their lives. Singh et al. [42] reported that patients with stable chronic schizophrenia whose continuing psychopathology was in the form of hallucinations, held a largely negative attitude towards hallucinations (negative, 45 to 94%; positive, 0 to 14%). 

In our study, mean duration of illness in nonaffective group was longer compared to affective group, and probably this led to significant negative content of voices and distress associated with it. The mean frequency of auditory hallucination in our study was 2.11 ± 1.86 which is higher than that of an earlier study [53]. The hypothesis that believability in hallucinations (i.e., the degree of conviction in the validity of hallucinations) at least partially mediates the positive relationship between the frequency of hallucinations and the distress associated with it [54] is also validated in our study. Few previous studies [15, 53] reported that patients who develop schizophrenia initially experience benign, nonclinically relevant AVHs, the content of which then becomes negative and distressing as a result of a traumatic/abusive experience, and the possibility of previous finding in our study cannot be ruled out in which there is significant finding in negative content of the voice and distress associated with it in nonaffective group. AVHs with benign content can become negative and distressing due to broader psychosocial factors affecting the individual [55].

The role of emotional factors in the experience of AVHs is widespread. Three aspects can be distinguished: emotional antecedents, emotional content, and emotional consequences. AVHs have often a negative, maladaptive quality in nonaffective disorder. Voices may insult and criticize the patient, tell the patient to do something unacceptable (e.g., to commit suicide or to harm someone), or threaten the patient. All these reasons were there in our study, and they also lead to significant finding in emotional characteristics in nonaffective group. The content of voices (e.g., as persecutory, abusive, obscene, derogatory, guiding, affirming, inspiring, threatening, etc.), is usually found in nonaffective group. Most predominant pervasive mood in nonaffective group in our study was indifferent and irritable. In affective group only in 16 patients the pervasive mood was euphoric and in remaining 14 patients the pervasive mood was irritable (*n* = 11), anxious (*n* = 2), and depressed (*n* = 1). However, in the 16 patients with euphoric mood the presence of secondary delusion, particularly persecution, decreased their intensity of euphoria. Copolov et al. [56] examined the affective impact of AVHs in a group of 199 patients (the majority with schizophrenia and affective psychosis) and found similar type of affect as in our study. Cheung et al. [57] also identified a vast range of emotional responses to voices. These included terror, irritation, sadness, and confusion. The most often reported response was anger and anxiety. However, the third most often reported emotion was happiness, which was reported by 29% of their participants. 

We found that those who indicated disruption had stronger feelings about their Ahs, and the greater the disruption, the greater the negativity with which they are evaluated. The negative content and the affect associated with it cause a lot of distress, and this leads to difficulty in performing the task with obvious disruption in the behaviour. This is the reason for getting significant differences in disruption item in nonaffective disorder patients. This finding is similar to the finding of Jenner et al. [49]. In another study, 75 percent of patients described being disrupted “moderately” or “a lot” and, on average, they obtained clearly negative scores [58].

### 5.3. Total Score

Mean total auditory hallucinations scores in affective and nonaffective groups were 11.80 ± 15.05 and 24.63 ± 15.89, respectively, and the difference was statistically significant (Table 3). It indicates that auditory hallucination was prominent psychopathology in nonaffective group population. Median severity score (sum of all the items of AH) in the study by Haddock et al. [30] was 28 but in that study, 73% of patients had a diagnosis of schizophrenia, while 27% belonged to the category of schizoaffective disorders. In contrast to the present study though 83.3% patients had schizophrenia, the rest had other psychoses excluding schizoaffective disorder which could account for the lower scores. It has been estimated that approximately 75% of the people with schizophrenia experience auditory hallucinations [46]. In our study, 73% of the patients have auditory hallucination in nonaffective group. Voices that comment on or discuss the individual's behaviour and that refer to the patient in the third person are included in Schneider's first-rank symptoms [59] and of diagnostic significance for schizophrenia. Studies show that approximately half of patients with schizophrenia experience these symptoms [60]. So the diagnosis of schizophrenia usually hovers around hallucinatory phenomena and our study is no exception.

### 5.4. Insight into Psychosis

Nearly 60% of the patients with schizophrenia and nearly 50% of the subjects with manic depression (with psychosis) are unaware of being ill [61]. Relationships have been noted between poor insight, clinical symptoms, and cognitive impairments. The presence of auditory hallucination, inappropriate affect, delusions, and thought disorder showed the most significant impact on insight levels. In our study, there was significant difference between affective and nonaffective groups in total score and self-certainty subscale of the BCIS. Self-certainty subscale measures decision-making and resistance to feedback. The finding of negative correlation between BCIS-total and total auditory hallucinations scores was explained by the fact that items like amount of negative content of voices, degree of negative content of voices, amount of distress, intensity of distress with voice, and emotional characteristic subscale score of AH contributed to the compromise of the insight. All these also lead to loosening of “normal” everyday associations and difficulties with reasoning. Thus it may not be surprising that when these symptoms are present patients have deficient awareness of their illness. Nayani and David [46] found a weak relationship between poor insight and the presence of hallucinations, suggesting that patients with hallucinations are more aware that this symptom is a deviation from the norm or find it relatively easy to relabel them but in our study voices were more distressing and disruptive to cope with. Lera et al. [62] concluded that patients with persistent hallucinations showed significantly less insight than patients without persistent hallucinations which is in agreement with our findings. Patients having acute [63] as well as those with chronic conditions [64, 65] show significant correlations between positive rather than negative symptoms and poor insight. Our study also supports the previous finding. In other studies, however, thought disorder and delusions were the most prominent positive symptoms, plus inappropriate affect (which may be classified as a facet of disorganization) which, when present, were associated with poor insight [64, 66–69] that was different from our study in which hallucination was most prominent and distressing. So higher insight is associated with less global psychopathology which is the usual finding in most studies [70, 71].

## 6. Conclusion

Hallucinating patients, more in nonaffective group, described their auditory hallucination as distressing both in terms of amount and intensity. Amount and degree of negative content of voices touch emotional aspect of the patients in nonaffective group. Significant differences in total score and Self-certainty subscale of BCIS were found but with negative correlation between BCIS-total and total auditory hallucinations scores.

## Figures and Tables

**Table 1:** Comparison of demographic variables of patients of affective and nonaffective groups.

Variable	Groups		*t*/*χ* ^2^	df	*P *
	Affective (*N* = 30) mean ± SD *n* (%)	Nonaffective (*N* = 30) mean ± SD *n* (%)			
Age (years)	33.43 ± 11.29	35.36 ± 9.23	0.72	58	0.471
Age of onset (years)	26.00 ± 7.55	31.13 ± 9.49	2.31	58	0.024*
Duration of illness (days)	84.50 ± 78.67	1338.23 ± 1815.40	3.77	58	0.000*
Duration of untreated psychosis (days)	79.13 ± 67.19	1168.40 ± 1679.63	3.54	58	0.001*
Duration of treated psychosis (days)	5.36 ± 17.75	169.83 ± 607.68	1.48	58	0.144
Sex					
Male	22 (73)	22 (73)	0.00	1	1
Female	8 (27)	8 (27)			
Religion					
Hindu	27 (90)	25 (83)	0.577	1	0.448
Muslim	3 (10)	5 (17)			
Marital status					
Single	5 (17)	4 (13)	0.131	1	0.718
Married	25 (83)	26 (87)			
Education					
Illiterate	11 (37)	11 (37)	2.073	2	0.355
Up to matriculation	18 (60)	15 (50)			
Above matriculation	1 (3)	4 (13)			
Community					
Tribal	2 (7)	2 (7)	0.000	1	1.000
Nontribal	28 (93)	28 (93)			
Mother tongue					
Hindi	26 (87)	21 (70)	2.532	2	0.282
Urdu	3 (10)	6 (20)			
Others	1 (3)	3 (10)			
Habitat					
Rural	25 (83)	29 (97)	2.963	1	0.085
Nonrural	5 (17)	1 (3)			
Occupation					
Unemployed	11 (37)	15 (50)	1.425	2	0.490
Laborer	4 (13)	2 (7)			
Others	15 (50)	13 (43)			
Economic status					
Lower	22 (73)	19 (63)	0.693	2	0.405
Middle	8 (27)	11 (37)			

*Significant.

**Table 2:** Clinical characteristics of the patients of affective and nonaffective groups.

Variables	Groups		*χ* ^2^	df	*P *
	Affective (*N* = 30) *n* (%)	Nonaffective (*N* = 30) *n* (%)			
Stressor					
Present	7 (23)	9 (30)	0.341	1	0.559
Absent	23 (77)	21 (70)			
Onset					
Acute	21 (70)	2 (7)	25.452	1	0.000*
Insidious	9 (30)	28 (93)			
Course					
Episodic	21 (70)	5 (17)	17.376	1	0.000*
Continuous	9 (30)	24 (80)			
Fluctuating	0 (0)	1 (3)			
Progress					
Deteriorating	30 (100)	29 (97)	0.000	1	1.000
Static	0 (0)	1 (3)			
Treatment history					
Yes	14 (47)	11 (37)	0.617	1	0.432
No	16 (53)	19 (63)			
Past history					
Yes	22 (73)	5 (17)	19.461	1	0.000*
No	8 (27)	25 (83)			
Family history					
Yes	10 (33)	15 (50)	1.714	1.0	0.190
No	20 (67)	15 (50)			
Degree of relationship					
First degree	9 (30)	8 (27)	5.273	2	0.072
Other	1 (3)	7 (23)			
No	20 (67)	15 (50)			
Support					
Adequate	29 (97)	29 (97)	0.000	1	1.000
Inadequate	1 (3)	1 (3)			
Number of episodes					
1	1 (3)	25 (83)	39.871	4	0.000*
2	7 (23)	1 (3)			
3	13 (43)	2 (7)			
4	5 (17)	1 (3)			
5	4 (13)	1 (3)			
Form of voice					
Nil	17 (57)	8 (27)	5.554	1	0.018*
2nd person and 3rd person	13 ^ a^(43)	22 (73)			

*Significant.

^a^Only 2nd person.

**Table 3:** Comparison of the auditory hallucinations items of the psychotic symptom rating scale (PSYRATS) in affective and nonaffective groups.

Scales	Groups		*U *	*P *
	Affective (*N* = 30) mean ± SD	Nonaffective (*N* = 30) mean ± SD		
PANSS (hallucination)^a^	2.70 ± 2.01	4.06 ± 1.92	298.0	0.016*
AH frequency	1.50 ± 1.79	2.73 ± 1.74	282.0	0.007*
AH duration	1.40 ± 1.73	2.53 ± 1.71	295.5	0.015*
AH location	1.36 ± 1.77	2.43 ± 1.73	304.0	0.021*
AH loudness	1.20 ± 1.49	2.10 ± 1.44	301.0	0.020*
AH beliefs regarding origin of voices	1.16 ± 1.51	2.16 ± 1.59	299.5	0.019*
AH amount of negative content of voices	0.66 ± 1.21	2.13 ± 1.50	224.5	0.000*
AH degree of negative content	0.60 ± 1.91	2.20 ± 1.56	214.5	0.000*
AH amount of distress	0.90 ± 1.39	2.26 ± 1.57	248.0	0.002*
AH intensity of distress	6.93 ± 1.46	2.13 ± 1.61	248.0	0.001*
AH disruption	0.83 ± 1.28	2.00 ± 1.57	266.0	0.003*
AH control	1.20 ± 1.66	2.06 ± 1.74	326.0	0.050
AH total score	11.80 ± 15.05	24.63 ± 15.89	257.0	0.003*
AH emotional characteristic	3.13 ± 5.06	8.73 ± 5.98	231.0	0.001*
AH cognitive interpretation	3.20 ± 4.16	6.10 ± 4.35	292.5	0.015*
AH physical characteristic	5.06 ± 6.35	9.80 ± 6.32	279.5	0.009*

AH: auditory hallucination; ^a^hallucinatory behavior subscales of positive and negative syndrome scale (PANSS); *significant.

**Table 4:** Comparison of the delusion items of the psychotic symptom rating scale (PSYRATS) in affective and nonaffective groups.

Scales	Groups		*U *	*P *
	Affective (*N* = 30) mean ± SD	Nonaffective (*N* = 30) mean ± SD		
PANSS (delusion)^a^	4.93 ± 1.14	4.40 ± 1.61	364.0	0.122
D amount of preoccupation	3.60 ± 1.13	3.16 ± 1.51	375.0	0.126
D duration of preoccupation	3.60 ± 1.06	3.16 ± 1.51	387.0	0.208
D conviction	3.63 ± 1.06	3.03 ± 1.49	333.5	0.027*
D amount of distress	1.90 ± 1.84	2.56 ± 1.69	368.5	0.200
D intensity of distress	1.86 ± 1.94	2.63 ± 1.71	364.0	0.168
D disruption	2.13 ± 1.81	2.63 ± 1.65	388.0	0.331
D total score	16.73 ± 6.87	17.20 ± 8.75	414.5	0.590
D emotional characteristic	6.16 ± 14.42	5.20 ± 3.39	376.0	0.247
D cognitive interpretation	12.96 ± 4.02	12.00 ± 5.77	449.5	0.994

D: delusion; ^a^delusion subscales of positive and negative syndrome scale (PANSS); *significant.

**Table 5:** Comparison of the beck cognitive insight scale (BCIS) and brief psychiatric rating scale (BPRS) scores in affective and nonaffective groups.

Scales	Groups		*U *	*P *
	Affective (*N* = 30) mean ± SD	Nonaffective (*N* = 30) mean ± SD		
BPRS	47.16 ± 9.00	39.83 ± 6.67	202.0	0.000*
BCIS self-reflectiveness	16.13 ± 4.07	15.43 ± 3.29	389.5	0.369
BCIS self-certainty	15.13 ± 2.78	12.80 ± 1.86	210.0	0.000*
BCIS total	31.26 ± 5.29	28.33 ± 4.49	280.0	0.012*
BCIS composite index	1.00 ± 4.55	2.53 ± 2.92	396.0	0.421

*Significant.
